# Comparison of the Anti-bacterial Efficacy of *Saussurea costus* and *Melaleuca alternifolia* Against *Porphyromonas gingivalis, Streptococcus mutans*, and *Enterococcus faecalis*: An *in-vitro* Study

**DOI:** 10.3389/froh.2022.950840

**Published:** 2022-06-27

**Authors:** Munerah S. BinShabaib, Shatha S. ALHarthi, Bashayer S. Helaby, Manar H. AlHefdhi, Afrah E. Mohammed, Kawther Aabed

**Affiliations:** ^1^Preventive Dental Sciences, College of Dentistry, Princess Nourah Bint Abdulrahman University, Riyadh, Saudi Arabia; ^2^College of Dentistry, Princess Nourah Bint Abdulrahman University, Riyadh, Saudi Arabia; ^3^Biology Department, College of Sciences, Princess Nourah Bint Abdulrahman University, Riyadh, Saudi Arabia

**Keywords:** *Saussurea costus*, *Melaleuca alternifolia*, tea tree oil, essential oil, antibacterial

## Abstract

The aim was to compare the *in-vitro* antibacterial effectiveness of two herbal extracts (a) *Saussurea-costus* (*S. costus*) and (b) *Melaleuca-alternifolia* (*M. alternifolia*) against *Porphyromonas gingivalis* (*P. gingivalis*), *Streptococcus mutans* (*S. mutans*) and *Enterococcus faecalis* (*E. faecalis*). Aqueous extracts from *M*. *alternifolia* were prepared by adding 2 grams of *S. costus* and *M. alternifolia*, respectively to 100 ml distilled water. Bacterial strains of *P. gingivalis, E. faecalis* and *S. mutans* were treated into 3 groups. In groups 1 and 2, bacterial strains were treated with aqueous extracts of *S. costus* and *M. alternifolia*, respectively. In the control-group, bacterial strains were exposed to distilled water. Antibacterial activity of the samples and nanoparticles was determined. The minimum-inhibitory-concentration (MIC) values were determined using the microdilution method. *P* < 0.01 was considered statistically significant. The MIC for all bacterial strains treated with *S. costus* was significantly higher than that of *M*. *alternifolia* (*P* < 0.001). There was no significant difference in MIC for strains of *P. gingivalis, E. faecalis* and *S. mutans* treated with *S. costus*. For bacterial strains treated with *M*. *alternifolia*, the MIC was significantly higher for *P. gingivalis* compared with *E. faecalis* and *S. mutans* strains (*P* < 0.01). There was no difference in MIC for *E. faecalis* and *S. mutans* strains treated with *M*. *alternifolia*. The *in-vitro* antibacterial efficacy of *M. alternifolia* is higher than *S. costus* against *P. gingivalis, E. faecalis* and *S. mutans*.

## Introduction

“Phytotherapy” is a term that is used in the field of medicine in which, either herbs or their extracts are used as either health promotion agents or to treat diseases [1]. This form of therapy encompasses Ayurvedic medicine, anthroposophic medicine, and traditional Chinese medicine. Herbs and their extracts such as the *Salvadora persica* chewing stick or “miswak” and Azadirachta indica or “neem” are often in the form of toothpastes and oral rinses for routine oral hygiene maintenance to eradicate dental plaque from teeth surfaces [2, 3]. In addition, these natural products are also known to exhibit antibacterial action against pathogenic bacteria such as *Streptococcus mutans* (*S. mutans*), *Porphyromonas gingivalis* (*P. gingivalis*), and *Enterococcus faecalis* (*E. faecalis*) [4–9]. Although numerous studies have investigated the bactericidal efficacy of herbal extracts of neem and miswak; other natural products that have been reported to exhibit antibacterial properties include *Saussurea costus* (*S. costus*) and *Melaleuca alternifolia* (*M*. *alternifolia*) [10, 11].

The *S. costus* (India costus), is a member of the *Asteraceae* family and is commonly used in countries such as Saudi Arabia, and India for different medical issues such as asthma, breast and hepatic cancer and thyroid diseases [12–14]. It is also known as *costus, putchuk*, or *kuth*. Likewise, *M*. *alternifolia*, also known as “tea tree oil” exhibits antimicrobial effects against microbes including *Staphylococcus aureus, S. mutans* and *Candida albicans* [15–18]; and promotes wound healing [18]. It is well-known that pathogenic microbes play a critical part in etiopathogenesis of dental caries and periodontal and peri-implant diseases [19–22]. The authors stringently reviewed indexed literature and observed that to date, there are no clinical and/or experimental studies that have assessed the antibacterial efficacy of *S*. *costus* and *M*. *alternifolia* against oral pathogenic bacteria. We hypothesize that *S. costus* and *M*. *alternifolia* exhibit bactericidal effects against *P. gingivalis, S. mutans* and *E. faecalis* with no difference in efficacy between the two herbals.

With this background, the objective was to *in vitro* investigate the antibacterial effectiveness of *S. costus* and *M. alternifolia* against *P. gingivalis, S. mutans*, and *E. faecalis*.

## Methods

### Ethical Statement and Informed Consent

Ethical approval was obtained from the Research Ethics Review Committee of the Princess Nourah Bint Abdulrahman University, ArRiyadh, Riyadh, Saudi Arabia. This is a laboratory-based investigation; therefore, the study was exempted from a written informed consent requirement.

### Sample Collection and Preparation of Aqueous Extracts

Samples of *S. costus* and *M. alternifolia* were purchased from a local-market in Riyadh, Saudi-Arabia. The samples were rinsed with distilled water, air-dried, and ground into a fine powder using a milling machine (IKA Werke Laboratory Equipment, Staufen, Germany). The milled materials were stored at room temperature in sealed plastic boxes until further analysis. Aqueous extracts from *M*. *alternifolia* were prepared by adding 2 grams of *S. costus* and *M. alternifolia*, respectively to 100 ml distilled water. Heat treatment was performed on the aqueous extract for 15 min at 80°C to stop the enzyme activity. The samples were filtered (Whatman candidate #1 pore size 125mm, Whatman, Maidstone, United Kingdom) and stored at 4°C till use. All samples were assessed within 24 h.

### Study Groups and Evaluation of Antibacterial Activity

Bacterial strains of *P. gingivalis, E. faecalis* and *S. mutans* were therapeutically classified into 3 groups. In groups 1 and 2, bacterial strains were treated with aqueous extracts of *S. costus* and *M. alternifolia*, respectively. In the control-group, bacterial strains were exposed to distilled water. Antibacterial activity was assessed using well agar diffusion method [23]. Three types of bacterial strains, *P. gingivalis* (ATCC^®^ 53978), *E. faecalis* (ATCC^®^ 29212) and *S. mutans* (ATCC^®^ 55676) were used. Microbial cultures were sub-cultured on Mueller-Hinton-Agar. 0.2 ml of bacteria strain (1.5 X 108 CFU/ml) was uniformly swabbed on agar plates using sterile-swabs; and 3 spaced wells (each 4 mm in diameter) were made per plate at the culture agar surface using a sterile metal-cork borer. In each well, 0.2 ml of extract was placed under aseptic conditions, and kept at room temperature for 1 h. Sterile distilled water was used as the negative reference control. Plates were incubated at 37°C for 24-h. Inhibition zones, which appeared as a clear area around the wells were evaluated.

### Assessment of MIC

The minimum inhibitory concentration (MIC) values were determined *via* microdilution method as described by Arends et al. [24]. In summary, 10 ml of a bacterial strain containing 1.5 x 108 colony forming units per milliliter (CFU/ml) was used. Different concentrations of nanoparticles were added to the test tubes containing the bacterial strains and incubated for 24 h. After incubation, the MIC values were obtained by checking the turbidity of the bacterial growth. The MIC value corresponded to the concentration that inhibited 99% of bacterial growth.

### Statistical Analysis

Statistical comparisons were done using a software (SPSS 20, Chicago, I.L. United States). Group comparisons in terms of reduction in CFU/ml in the study groups was done using the one-way analysis of variance. For multiple comparisons, the Bonferroni *post-hoc* correction test was also performed. *P*-values below 0.01 were considered statistically significant.

## Results

### Antibacterial Activity of *S. costus* and *M. alternifolia*

**Figure 1:**
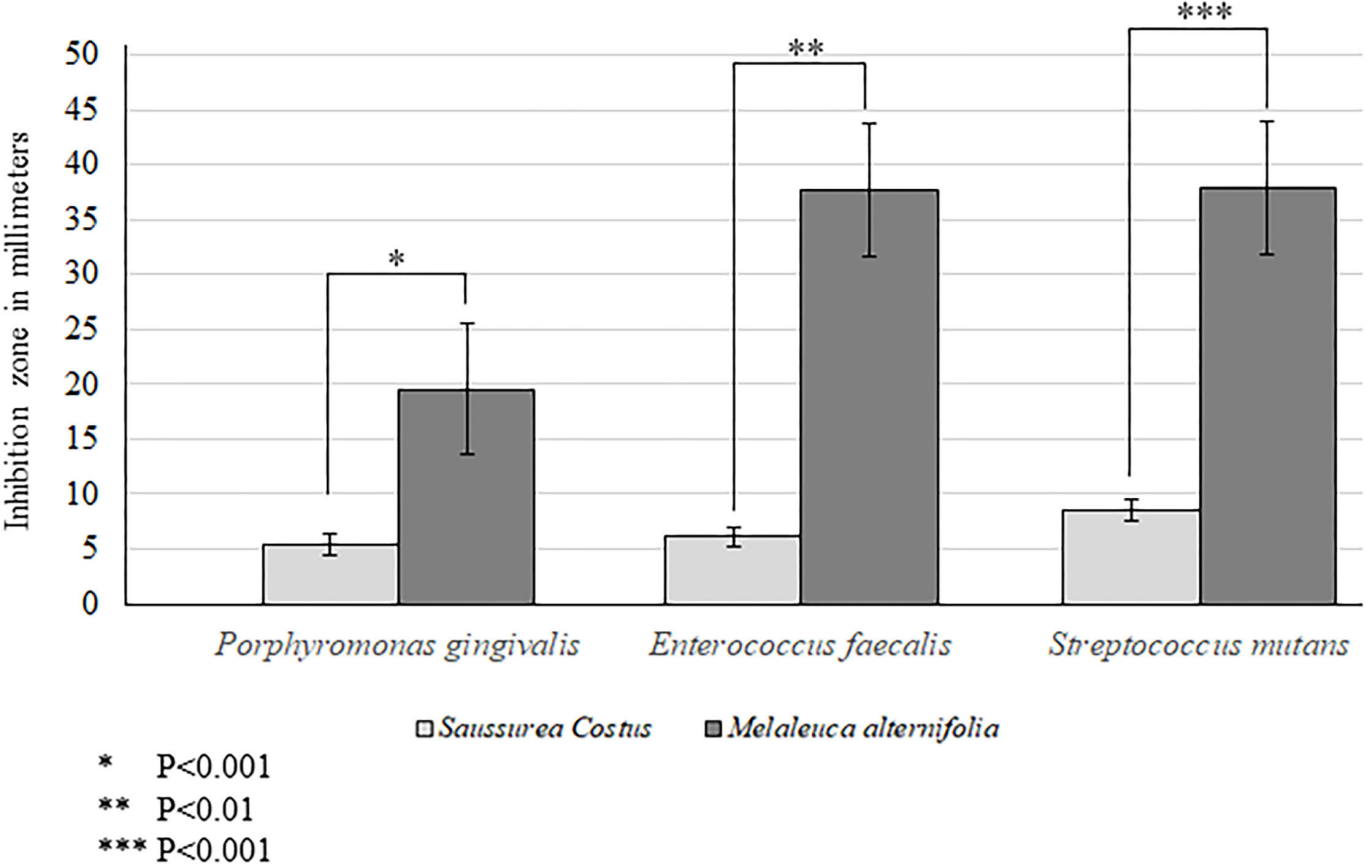
Antibacterial efficacy of *Melaleuca alternifolia* and *Saussurea costus*.

Aqueous extracts of *M. alternifolia* demonstrated a significantly high antimicrobial activity against strains of *P. gingivalis* (*P* < 0.001), *E. faecalis* (*P* < 0.01) and *S. mutans* (*P* < 0.001) compared with extracts of *S. costus* (Figure 1). The antibacterial activity of *M. alternifolia* was significantly higher against *E. faecalis* and *S. mutans* compared with that against *P. gingivalis*. There was no significant difference in the antibacterial efficacy of *M. alternifolia* against *E. faecalis* and *S. mutans* (Figure 1). There was no bactericidal activity observed in the control-group compared with strains that were treated with *S*. *costus* and *M. alternifolia*.

### MIC of *S. costus* and *M. alternifolia* in Relation to Their Bactericidal Efficacy

The MIC for all bacterial strains treated with *S. costus* was significantly than that of *M*. *alternifolia* (*P* < 0.001). There was no statistically significant difference in the MIC for strains of *P. gingivalis, E. faecalis* and *S. mutans* treated with *S. costus*. For bacterial strains treated with *M*. *alternifolia*, the MIC was significantly higher for *P. gingivalis* compared with *E. faecalis* and *S. mutans* strains (*P* < 0.01). There was no significant difference in the MIC for *E. faecalis* and *S. mutans* strains treated with *M*. *alternifolia*. The MIC for *S. costus* and *M. alternifolia* are shown in Table 1.

**Table 1:** Minimum inhibitory concentrations of *Saussurea costus* and *Melaleuca alternifolia* in relation to their bactericidal efficacy.

**Bacterial strain**	***Porphyromonas gingivalis***	***Enterococcus faecalis***	***Streptococcus mutans***
*Saussurea costus*	56.2 ± 9.6 g/ml^*^	56.6 ± 7.6 g/ml^*^	80.5 ± 10.2 g/ml^*^
*Melaleuca alternifolia*	29.5 ± 1.6 g/ml	13.5 ± 2.5 g/ml^†^	15.5 ± 3.7 g/ml^†^

* *Compared with the same bacterial strain treated with M. alternifolia (P < 0.001)*.

† *Compared with P-gingivalis strain treated with M. alternifolia (P < 0.01)*.

## Discussion

It has already been reported that *S. costus* and *M*. *alternifolia* possess antibacterial effects [9]; however, to the authors knowledge from pertinent indexed literature, the present study is the first one to compare the antibacterial efficacy of *S. costus* and *M. alternifolia* against common oral pathogenic bacteria (*P. gingivalis, E. faecalis* and S. mutans). The present experiment was based on the hypothesis that *S. costus* and *M*. *alternifolia* exhibit bactericidal effects against *P. gingivalis, S. mutans* and *E. faecalis* with no difference in efficacy between the two herbals. The results of the present experiment are partially in agreement with the proposed hypothesis as both herbal extracts (*S. costus* and *M*. *alternifolia*) demonstrated antibacterial efficacy against the tested bacterial strains. However, this experiment showed that antibacterial effectiveness of *M*. *alternifolia* was superior to that of *S. costus* in terms of MIC and identification of inhibition zone. It is challenging to determine the precise factor/s that may have contributed in this regard; however, different theories may be proposed. Firstly, tea tree oil or *M*. *alternifolia* is a well-known anti-inflammatory and antiseptic agent. Moreover, *M*. *alternifolia* inhibits bacterial respiration and disrupts permeability barrier of microbial cell membrane [17]; and at the same time increases the leakage of potassium ions in both Gram-negative and -positive bacteria [17]. These are possible factors that may be associated with the superior antibacterial effectiveness of *M*. *alternifolia* over *S. costus*. The current experiment showed that the MIC was significantly higher for *P. gingivalis* compared with *E. faecalis* and *S. mutans* strains for bacterial strains treated with *M*. *alternifolia*. One justification for this is that a standard concentration of *M*. *alternifolia* of 0.2% such as that used in the present investigation is insufficient to demonstrate antibacterial effectiveness against all types of bacteria. It is therefore hypothesized that *M*. *alternifolia* with used in concentrations higher than 0.2% demonstrates antibacterial effectiveness and creates inhibition zones that are statistically similar to those created for other bacteria such as *E. faecalis* and *S. mutans*. Further studies are needed to test this hypothesis.

From a clinical perspective, it is well-known that periodontopathogenic bacteria such as *P. gingivalis* play a role in the etiopathogenesis and progression of oral inflammatory conditions such as periodontitis [25–27]. Traditionally, mechanical instrumentation of periodontal sulci and related teeth/root surfaces is performed for the management of periodontitis [25]; and postoperative oral rinses such as 0.12% Chlorhexidine gluconate (CG) are often prescribed to patients in order to facilitate healing. However, this protocol is difficult to adopt particularly in patients with chlorhexidine allergy (CA). Although CA is a rare condition, it may be a source of distress to patients as it induces type-IV hypersensitivity reactions such as erythema in gingival tissues, burning sensation in the mouth and stomatitis [28, 29]. In a recent randomized controlled trial (RCT), Al-Zawawi et al. [30] investigated the postoperative anti-inflammatory efficacy of herbal-based oral rinses after non-surgical instrumentation for the management of periodontal inflammation in patients with chlorhexidine allergy. The results showed that herbal-based oral rinses are suitable substitutes to 0.12% CG as post-operative prescriptions following periodontal treatment [30]. The present *in-vitro* results applaud the results reported by Al-Zawawi et al. [30]; and suggest that *S. costus* based mouthwashes can be used for the management of periodontal inflammatory conditions as a substitute to 0.12% CHX. However, this needs additional well-designed and power adjusted RCTs.

A major limitation of the present study is that the results were entirely based on *in-vitro* evaluation of aqueous extracts from both herbs. Several clinical studies have shown that habits such as tobacco smoking and systemic diseases including poorly-controlled diabetes mellitus are risk factors of periodontitis and dental caries [31–38]. Such risk-factors are known to compromise outcomes of oral therapeutic interventions [39]; and promotion of microbial colonization in the supra- and subgingival oral biofilm [40, 41]. In this context, it remains debatable whether or not periodontal therapy (surgical or non-surgical) and restoration of carious teeth with adjunct use of oral rinses derived from *S. costus* and *M*. *alternifolia* is effective in the restoration of a clinically acceptable oral health status. Further studies, predominantly RCTs are needed.

## Conclusion

*In-vitro* antibacterial efficacy of *M. alternifolia* is higher than *S. costus* against *P. gingivalis, E. faecalis* and *S. mutans*. From a clinical perspective, it is speculated that oral dentifrices based on *M. alternifolia* extracts can play a role in the maintenance of oral health and treatment of oral diseases such as periodontitis.

## Data Availability Statement

The original contributions presented in the study are included in the article/supplementary materials, further inquiries can be directed to the corresponding author/s.

## Author Contributions

MB and SA: study design and manuscript writing and revisions. BH: methodology and manuscript writing and editing. MA: statistical analysis, methodology, and manuscript writing and editing. AM: methodology and manuscript writing and editing. KA: supervision, methodology, and manuscript writing and revisions. All authors contributed to the article and approved the submitted version.

## Conflict of Interest

The authors declare that the research was conducted in the absence of any commercial or financial relationships that could be construed as a potential conflict of interest.

## Publisher's Note

All claims expressed in this article are solely those of the authors and do not necessarily represent those of their affiliated organizations, or those of the publisher, the editors and the reviewers. Any product that may be evaluated in this article, or claim that may be made by its manufacturer, is not guaranteed or endorsed by the publisher.
